# Non-Supported and PET-Supported Chitosan Membranes for Pervaporation: Production, Characterization, and Performance

**DOI:** 10.3390/membranes12100930

**Published:** 2022-09-25

**Authors:** Wendel Paulo Silvestre, Jocelei Duarte, Isabel Cristina Tessaro, Camila Baldasso

**Affiliations:** 1 Postgraduate Program in Chemical Engineering, Federal University of Rio Grande do Sul, Porto Alegre 90010-150, Brazil; 2 Postgraduate Program in Process Engineering and Technologies, University of Caxias do Sul, Caxias do Sul 95070-560, Brazil

**Keywords:** biopolymer, pervaporation membranes, composite membranes, sorption–diffusion

## Abstract

The objective of this study was to develop non-supported and PET-supported chitosan membranes that were cross-linked with glutaraldehyde, then evaluate their physical–chemical, morphological, and mechanical properties, and evaluate their performance in the separation of ethanol/water and limonene/linalool synthetic mixtures by hydrophilic and target-organophilic pervaporation, respectively. The presence of a PET layer did not affect most of the physical-chemical parameters of the membranes, but the mechanical properties were enhanced, especially the Young modulus (76 MPa to 398 MPa), tensile strength (16 MPa to 27 MPa), and elongation at break (7% to 26%), rendering the supported membrane more resistant. Regarding the pervaporation tests, no permeate was obtained in target-organophilic pervaporation tests, regardless of membrane type. The support layer influenced the hydrophilic pervaporation parameters of the supported membrane, especially in reducing transmembrane flux (0.397 kg∙m^−2^∙h^−1^ to 0.121 kg∙m^−2^∙h^−1^) and increasing membrane selectivity (611 to 1974). However, the pervaporation separation index has not differed between membranes (228 for the non-supported and 218 for the PET-supported membrane), indicating that, overall, both membranes had a similar performance. Thus, the applicability of each membrane is linked to specific applications that require a more resistant membrane, greater transmembrane fluxes, and higher selectivity.

## 1. Introduction

Pervaporation is an emergent separation technique with great potential to be employed in several areas of research and industry [1]. Some studies have used pervaporation, especially organophilic pervaporation, to recover and obtain aroma compounds from the process streams of the food industry [2,3,4,5], pervaporation being acknowledged as an innovative and emerging separation process for these types of compounds [6].

Most pervaporation membranes are composed of at least two layers, one dense but generally very thin, being responsible for the separation process (active layer), and a support layer, thicker and porous, which supports the active layer. Sometimes, there is also a third layer (interlayer), which is applied when the polymers of the active and support layers have a low affinity, or to promote stronger interactions between the two layers [7,8].

Considering that the driving force in pervaporation is established through a partial vapor pressure difference (the application of a vacuum) in the membrane side opposite to the feed stream, the necessity for thin, active layers renders monolayer membranes quite fragile, hindering their capability to withstand mechanical strain during operation [9,10].

While pervaporation is regarded as an efficient, economic, and environmentally friendly process, in the long run, the efficiency of this process relies mostly upon membrane characteristics and performance [9,10,11]. Most of the membranes produced both in industry and research are composite membranes. This may ally the use of a very thin active layer enhancing membrane efficiency with the use of a robust support layer, capable of withstanding the conditions of the pervaporation process and providing protection to the active layer, especially in processes that have important variations in the operational conditions as to when the polymer of the active layer has poor mechanical characteristics, rendering it prone to failure [12,13,14].

Regarding the use of support layers to improve the mechanical properties of membranes, PET layers are widely used in several types of membrane separation processes, ranging from microfiltration to reverse osmosis and pervaporation. The mechanical properties, easy fabrication, and low cost make this type of support layer suitable for use in both circular and plane membrane configurations [15,16,17].

Chitosan is nowadays seen as one of the most important biopolymers in production, due to its unique versatility and capacity for employment in several areas of study, from medical uses, such as tissue scaffolds, to active packaging [18,19,20,21]. The high chemical resistance of chitosan makes it suitable for use in membrane production. Considering the properties and potential of chitosan, the use of this biopolymer as the main material or as an additive in membrane production has increased greatly in the last few years [22]. There are reports of studies of chitosan-based membranes being employed in reverse and forward osmosis, ultra- and nanofiltration, and pervaporation [22,23,24,25].

Most studies that employed chitosan-based membranes in pervaporation focused on hydrophilic pervaporation, especially in terms of the dehydration of alcohols [24,26,27,28,29]. The literature reports few studies that have employed chitosan in organophilic and target-organophilic pervaporation; relative to the latter, the most studied mixtures are binary, composed of an aromatic compound (benzene or toluene) and a polar compound (ethanol or methanol) [30,31,32,33]. In addition, most studies in the production and development of chitosan-based supported membranes do not address in depth the presence of a support layer and its effects on membrane performance.

Given that background, this work aimed to develop non-supported and PET-supported chitosan membranes, crosslinked with glutaraldehyde, evaluate their physical–chemical, morphological, and mechanical properties, and evaluate their performance in the separation of ethanol/water and limonene/linalool synthetic mixtures by hydrophilic and target-organophilic pervaporation, respectively.

## 2. Materials and Methods

### 2.1. Membrane Preparation

The membranes were prepared following the procedures described by Frick et al. [34], with modifications. The polymeric solution was prepared using an aqueous solution of 1.0% vol. acetic acid, 0.75 wt % of chitosan, and 5.0 wt % (relative to chitosan content) of glutaraldehyde as the crosslinking agent. In terms of chemicals, we used glacial acetic acid AR-grade (Merck), scaled chitosan derived from crab shells (Sigma-Aldrich, St. Louis, MI, USA; CAS number 9012-76-4) with a deacetylation degree of 83.2 ± 4.0% and an average molar mass of 842.5 kDa, and glutaraldehyde AR-grade 25% vol. (Merck, Darmstadt, Germany). The solution was stirred for 24 h, using a magnetic stirrer (Velp Scientifica, ARE, Usmate Velate, Italy). After the addition of the crosslinker, the solution was again vigorously stirred for 15 min; afterward, it was kept at rest for 30 min.

The dynamic viscosity of the polymeric solution was determined according to the ASTM D2196-18e1 standard [35], using a rotary viscosimeter (Brookfield^®^, LVDV-II + P, Toronto, Canada) with an S64 spindle and a rotation speed of 50 rpm. The measurement was carried out in triplicates. The polymeric solution presented a dynamic viscosity of 285 ± 10 mPa·s^−1^.

Twenty milliliters of the polymeric solution were spread on Teflon^®^-coated Petri dishes with a diameter of 11 cm. The solution-coated dishes were dried in an oven (De Leo, DL-AFD) with forced air circulation for 24 h at 35 ± 3 °C. After drying, the membranes were removed from the Petri dishes and stored at room temperature and relative humidity, sealed inside closed plastic bags and kept away from sunlight.

To produce the supported membranes the same procedure was followed, in which the support layer (a PET sheet with an average thickness of 90 µm) was put on the polymeric solution immediately before the casted solution was to be dried, removing any air bubbles that were present in the interface between the polymer and the solution. The PET sheets were obtained from reverse osmosis membranes that were discarded from the industry. The active layer was mechanically removed, and the PET supporting layer was cleaned, rinsed with distilled water twice, and air-dried before being used in the preparation of the supported membranes.

### 2.2. Characterization of the Polymeric Solution

The characterization of the properties of the polymeric solution was carried out by viscosimetry (inherent and specific viscosities) and the evaluation of the Huggins (k_H_) and Kraemer (k_K_) constants [36,37,38]. Both constants were determined according to the Huggins equation, as proposed by Costa et al. [39].

The determination of the reduced and inherent viscosities was carried out following the procedures described by Czechowska-Biskup et al. [37], at a temperature of 25.0 ± 0.05 °C. We used an Ubberholde number 1 viscosimeter made of borosilicate glass, with a capillary tube with a diameter of 0.53 mm. The viscosimeter was operated in a thermostatic bath (Q303SR26, Quimis, Brazil).

A buffer solution of acetic acid 0.20 M and sodium acetate 0.15 M was used as a solvent for chitosan since this solution is considered a standard solution for the evaluation of solubilized chitosan [37]. The reduced and inherent viscosities of the polymeric solution, composed of acetic acid 1.0% *v*/*v* (0.17 M) and chitosan, were also determined to verify the effect of the solvent on the stability and behavior of the chitosan solution used in the production of the membranes.

### 2.3. Membrane Characterization

Membrane’s chemical structure and thermal properties were carried out by Fourier-transform infrared spectroscopy (FTIR) using a Nicolet iS10 spectrometer and the thermogravimetric analysis (TGA) using a Shimadzu TGA-50 thermobalance. Both TGA and FTIR analyses were carried out according to the procedures described by Frick et al. [34]. The mechanical properties of thickness, elasticity modulus, elongation at break, and tensile strength were evaluated.

The thickness of the membranes was determined using a Mitutoyo SKU 60992 digital micrometer. Ten random points were measured in each membrane; the average thickness was calculated by the arithmetic mean of the measurements. The mechanical properties were determined following the ASTM D882-18 standard [40], using an EMIC DL2000 machine with a 30 N load cell. The starting spacing between claws was 50 mm, and the velocity was 50 mm·min^−1^.

The contact angle test was carried out following the ASTM D7490-13 standard [41], using distilled water, diiodomethane 99 %, ethanol 96 vol.% (AR-grade), linalool (AR-grade), and myrcene (AR-grade). The contact angle was measured using a DMC-FZ40 digital camera (Panasonic, São Paulo, Brazil), and the data was processed using the Surftens 4.7 software (OEG GmbH, Hessisch Oldendorf, Germany). The surface energy of the membranes was determined by the method proposed by Fowkes [42], following the procedures described by Kozbial et al. [43]. Distilled water and diiodomethane 99 % (AR-grade) were used as the test liquids.

The swelling test was performed by immersing the membranes in four different liquids: distilled water, 96 vol.% ethanol, linalool, and myrcene. The membranes were kept immersed in the liquid for 24 h at 20 ± 2 °C. Then, the samples were carefully removed, dried with absorbent paper, weighed (analytical balance AL500C, Marte, Brazil) and their thickness was measured. The percentage of swelling was calculated using the mass and thickness of the membranes before and after immersion.

The membrane microstructure was evaluated via scanning electron microscopy, according to the procedures described by Pavoni [15]. Membrane samples were cryogenically fractured using liquid nitrogen and then metalized, using a thin gold layer to increase their electrical conductivity, and measuring it with an SSX-550 scanning electron microscope (Shimadzu, Japan) operating at 15 kV, employing different approximation scales (50× to 5000×).

### 2.4. Pervaporation Tests

Pervaporation tests were carried out using a stainless-steel circular module with an internal diameter of 3.69 cm and an effective permeation area of 10.68 cm^2^; the feed vessel had a volume of 150 mL. Feed temperature was kept at 40 ± 2 °C, the feed flow rate was 2.0 mL·s^−1^, and the pressure downstream of the membrane (permeate stream) was kept at 40 ± 2 Torr (5.33 ± 0.27 kPa). An E2M5 two-stage high-vacuum rotary vane pump (Edwards, UK) was used. A cold trap, with a mixture of sodium chloride, water ice, and ethanol (−15 ± 5 °C), was used to condense the permeate stream.

The feed stream for hydrophilic pervaporation was composed of a mixture of 70 wt % ethanol (99 %, CAS 64-17-5, Merck, Darmstadt, Germany) and 30 wt % water (distilled and deionized water). For target-organophilic pervaporation, the feed stream consisted of a mixture containing 70 wt % limonene (≥ 95 %, CAS 5989-54-8, Merck, Germany) and 30 wt % linalool (≥ 95 %, CAS 126-91-0, Merck, Germany). Every pervaporation test was carried out for 8 h.

All pervaporation parameters were determined following the definitions and equations proposed by Baker et al. [44]. The activity coefficients (γ) for the components of the feed streams (liquid phase) were calculated using the PRO/II simulation software (Aveva, Cambridge, UK) at 313 K (40 °C) with the non-random two-liquid (NRTL) thermodynamic model. The calculated coefficients were compared with the data from Teixeira et al. [45] and the Dortmund Data Bank [46].

### 2.5. Chromatographic Analyses

The feed and permeate streams of hydrophilic pervaporation were analyzed by HPLC, following the procedures described by Souza et al. [47]. We used a Shimadzu LC-20AD HPLC system (Shimadzu Corporation, Kyoto, Japan). The system was equipped with a CMB-20A controller, an LC-20AD isocratic pump, a CTO-20A column oven, and a RID-20A refractive index detector. The column used was an Aminex^®^ HPX-87H (Bio-Rad Labs, Richmond, CA, USA) with dimensions of 300 mm × 7.8 mm and 9.0 µm particle size. The mobile phase was sulfuric acid at 5 mM, with a flow rate of 0.5 mL∙min^−1^. The oven temperature was kept at 60 °C. Each run was carried out for 35 min. Ethanol content was determined using a calibration curve in the range of 0–15 wt % (0–150 g∙kg^−1^). Water content was determined by difference analysis.

### 2.6. Experimental Design and Statistical Analysis

The experimental design was completely randomized, and each evaluation was carried out using five replicates. The treatments were the non-supported (chitosan only) and the PET-supported membranes. The results underwent Levene’s test (homogeneity of variances) and the Shapiro–Wilk (normality of residuals) test, followed by an analysis of variance (ANOVA). The means were compared using Tukey’s multiple range test at a 5% error probability (α = 0.05). The statistical analysis was carried out using the Statistica 12.5 software (StatSoft, Tulsa, OK, USA).

## 3. Results and Discussion

### 3.1. Characterization of the Polymeric Solution

**Figure 1:**
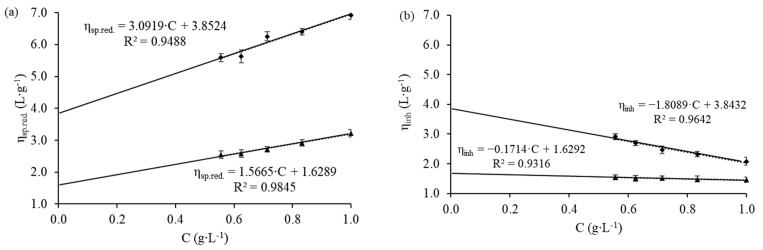
Reduced viscosity (**a**) and inherent viscosity (**b**) data for the buffer system of chitosan/acetic acid 0.20 M/sodium acetate 0.15 M (▲) and chitosan/acetic acid 1.00% *v*/*v* (0.17 M) (♦).

The reduced and inherent viscosities for the chitosan/acetic acid 0.20 M/sodium acetate 0.15 M and chitosan/acetic acid 1.00% *v*/*v* (0.17 M) buffer system were determined. The graphs compiling the results are presented in Figure 1.

The regressions, fitted to Huggins and Kraemer equations, yielded k_H_, k_K_, and k_H_ + k_K_ values of 0.590, −0.065, and 0.525, respectively, for the chitosan/acetic acid 0.20 M/sodium acetate 0.15 M buffer system. On the other hand, the behavior of the chitosan/acetic acid 1.00% *v*/*v* was quite different; the calculated k_H_, k_K_, and k_H_ + k_K_ values were 0.209, − 0.122, and 0.087, respectively.

Czechowska-Biskup et al. [37] reported that chitosan samples with an average molar mass of 477 kDa were then solubilized in the acetic acid 0.20 M/sodium acetate 0.15 M buffer system, with a k_H_ value of 0.538 and a k_K_ value of −0.071. According to Costa et al. [39], Czechowska-Biskup et al. [37], and Duarte et al. [38], k_H_ values in the range of 0.4 and k_H_ + k_K_ values in the range of 0.5 are indicators that the solution is thermodynamically stable; negative k_K_ values indicate that the polymeric chains of the chitosan solubilized well.

Although the negative k_K_ value indicates good solubilization of chitosan in the solution of acetic acid 1.00% *v*/*v* (0.17 M), the low k_H_ and k_H_ + k_K_ values are indicative that this system is thermodynamically unstable [39]. Thus, it is not advisable to store the polymeric solution; instead, it is recommended that you should prepare a fresh solution in each batch to avoid changes in the physical–chemical properties of the solution, during the production of the membranes.

### 3.2. Characterization of the Membranes

#### 3.2.1. Thermal Properties and Chemical Structure

**Figure 2:**
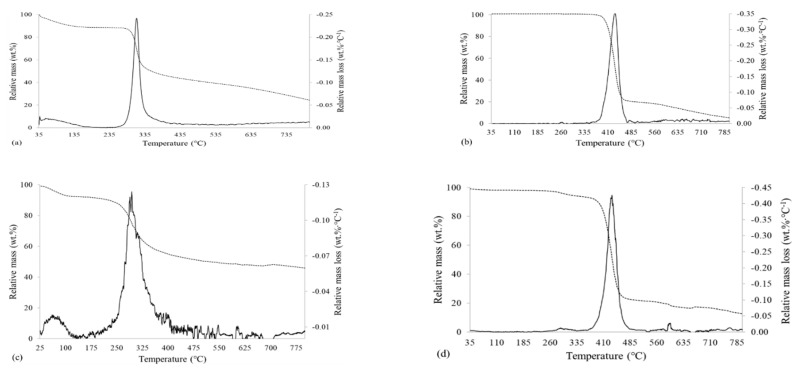
TGA (dashed line) and DTG (solid line) curves for chitosan (**a**), PET layer (**b**), non-supported membrane (**c**), and PET-supported membrane (**d**).

The TGA and DTG curves of chitosan, the PET support, and the supported and non-supported membranes are presented in Figure 2.

It was possible to observe from Figure 2a that, for chitosan, there was a region of slight mass loss of about 10 wt %, in the range of 50–150 °C, probably due to the volatilization of the remaining biomolecules from the production of chitosan, the start of the thermal degradation of smaller chains, and the removal of moisture from the material, the content of which was 9.90 wt % [34,48,49]. The highest mass loss, about 40 wt %, occurred in the range of 290–340 °C. In this temperature range, the highest depolymerization rate for chitosan was recorded. Other authors reported the peak depolymerization temperature for chitosan in the range of 305–320 °C [34,49,50]. Besides depolymerization, the cracking and dehydration reactions of the saccharide ring and decomposition of the acetyl groups may also have taken place [34,50].

The mass loss became progressive and roughly linear in the range of 340–800 °C, due to cracking, pyrolysis, and volatilization reactions of the polymeric chains of chitosan [49,50]. The remaining mass at 800 °C was 24.7 wt %; Fiori et al. [50] reported a remaining mass of 30 wt % at 600 °C, Matet et al. [49] observed about 35 wt % at 700 °C, and Frick et al. [34] reported, at 700 °C, that there was about 40 % of residual material.

Regarding the chitosan membrane—Figure 2c—it was possible to observe that both the TGA and DTG curves were similar to those of pure chitosan, as seen in Figure 2a. The higher mass loss that occurred in the range of 40–105 °C was probably the result of the dehydration of the membrane and volatilization of the remaining acetic acid, which could be captured by the membrane structure during the curing process. It was also possible to observe that the temperature range in which the highest mass loss of the membrane occurred was from 245 to 350 °C, this being wider than the range of pure chitosan (290–340 °C). Frick et al. [34], Matet et al. [49], and Fiori et al. [50] also reported similar phenomena, citing the disruptive effect of acetic acid on the structure of polymerized chitosan, even after crosslinking. As is the case with pure chitosan, the non-supported membrane also had a progressive and linear mass loss between 350 and 800 °C. The residual mass of the membrane at 800 °C was 45.9 wt %, being higher than the residual mass of pure chitosan (24.7 wt %), probably because of the crosslinker on the polymeric chains. Crosslinking may hinder the depolymerization of the chitosan structure, rendering difficult the generation of pyrolytic vapors that result from the cracking reactions [34].

The support—Figure 2b—had TGA and DTG curves that were quite different from the ones of pure chitosan—Figure 2a. This polymer started to degrade at approximately 380 °C, occurring a fast mass loss (about 75% of the starting mass) in the range of 380–480 °C, a result of the partial decomposition and volatilization of PET, considering that this polymer starts its thermolysis at 350–400 °C [51,52]. At 800 °C, only 5.6 wt % of the starting mass remained, indicating that this material produced smaller amounts of ash and other residues than chitosan (45.9 wt %).

The TGA and DTG curves of the supported membrane—Figure 2d—were similar to those of the support—Figure 2b—with some changes that are characteristic of the presence of the chitosan layer. A mass loss of about 3.0 wt % was observed at 250–305 °C, a result of the partial volatilization and decomposition of the active layer of crosslinked chitosan, occurring in the same temperature range of the non-supported membrane. The highest mass loss, about 70 wt %, occurred in the range of 380–470 °C, similar to the support. For the supported membrane, the maximum mass loss was at 434 °C. At 800 °C, the remaining mass of the supported membrane was 12.9 wt.%, whereas the one for the support layer of PET was 5.6 wt %, due to the presence of chitosan in the membrane, which presented a larger percentage of residual material at 800 °C (45.9 wt %).

Due to the observed behavior, there is probably no chemical reaction taking place between the chitosan active layer and the support layer. Thus, the adherence between them is the result of the drying process of the polymeric solution and the formation of contact points on the rough surfaces of the support.

**Figure 3:**
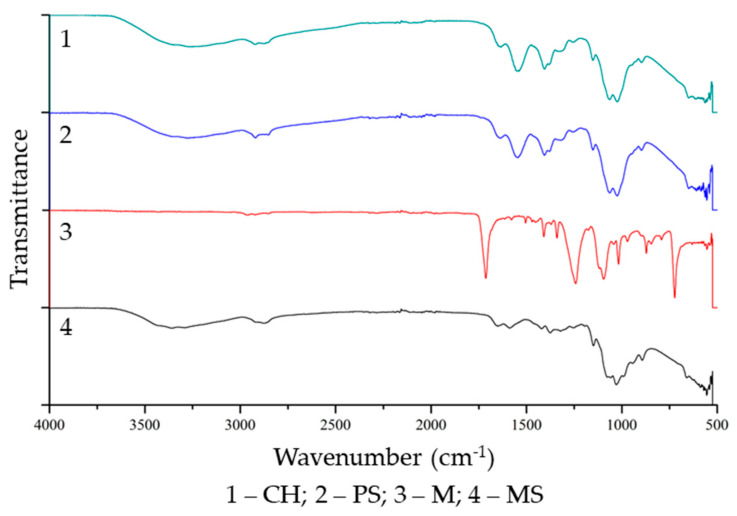
FTIR spectra of the chitosan (CH), the support (PS), and the non-supported (M) and supported (MS) membranes.

The FTIR spectra of chitosan, the support, and the supported and non-supported membranes are presented in Figure 3.

When analyzing Figure 3, it is possible to observe that the IR absorption spectra of chitosan (CH) and the supported (MS) and non-supported (M) membranes were similar, with the exception of the zones in the wavenumbers of 1650 cm^−1^ and 1400 cm^−1^, in which there was an increase in the absorption band of the membranes and whose absorption by chitosan was smaller. This range of wavenumbers corresponds to the absorption bands of carbonyl (C=O), N-H stretching, and primary and secondary amines; probably, the more intense absorption was the result of a reaction with glutaraldehyde during the crosslinking process [15,48,53,54].

**Figure 4:**
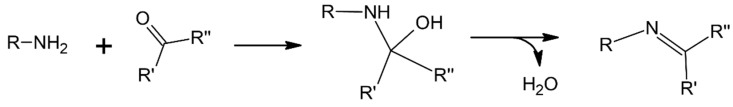
Crosslinking reaction between glutaraldehyde and chitosan, with the formation of an imine after membrane crosslink.

Frick et al. [34], in a similar study, pointed out that membrane crosslinking occurs due to a reaction between the formyl (-CHO) and amino (-NH_2_) functional groups, which are present in glutaraldehyde and chitosan, respectively. A simplified scheme of the crosslinking reaction between chitosan and glutaraldehyde is presented in Figure 4.

In Figure 4, R represents the saccharide ring of chitosan, R’ is the hydrogen atom of the formyl group, and R’’ is the rest of the glutaraldehyde molecule. The crosslinking generates a hemiaminal, which is dehydrated to an imine in membrane curing (water removal). The C=N bond (imine) has an IR absorption peak at approximately 1650 cm^−1^, indicating that the crosslinking of the membrane occurred with the formation of an imine between the crosslinker and the chitosan chains [34,48,54].

It was possible to observe the differences between the FTIR spectra of the supported membrane (M) and the support (PS); the IR absorption spectrum of the membrane was similar to that of chitosan (CH). This result indicates that the PET support layer was fully coated by the active chitosan layer and that there was no chemical reaction between the different membrane layers. Thus, the interaction between the active layer of chitosan and the support layer of PET is probably of physical origin, and no chemical reactions have taken place apart from the ones between the chitosan and the crosslinker. The occurrence of side reactions, especially between the active and support layers, may have a deleterious effect on overall membrane performance, such as a change in membrane permeability or the chemical affinity of the active layer with the components of the feed stream in the pervaporation process [7,8,10].

#### 3.2.2. Mechanical Properties

The evaluated mechanical properties of the PET-supported and non-supported membranes and of the support layer are compiled in Table 1.

Regarding the membrane thickness, the active layer of chitosan in the supported membrane (MS) was 28 ± 3 µm, while for the non-supported membrane (M), the layer thickness was 20 ± 1 µm, a difference of approximately 40%. It is important to mention that the same volume of polymeric solution (20 mL) was used in the casting of the membranes. The differences in thickness could be due to surface interaction effects between the PET layer and the Teflon^®^ of the Petri dishes. A capillary effect may have caused an accumulation of polymeric solution in the region between the polymers, which led to an increase in the overall membrane thickness concerning the non-supported membrane [34,55].

Frick et al. [34], who worked in the development of films and membranes using chitosan without a support and crosslinked with glutaraldehyde, reported thickness values in the range of 29–35 µm, considering zero and 10 wt % glutaraldehyde relative to chitosan mass, respectively. The same authors stated that a higher amount of crosslinker may alter the dynamic viscosity of the polymeric solution, which may cause unevenness during the curing of the newly formed membrane structure. Beppu et al. [56] reported the production of chitosan membranes crosslinked with glutaraldehyde, with an average thickness of 11 µm; however, these membranes were produced by casting, being posteriorly immersed in a glutaraldehyde solution for surface crosslinking. Zhang et al. [57] reported an average thickness of 20 µm for chitosan membranes crosslinked with glutaraldehyde in conditions that paralleled those in the present work.

The Young modulus of the supported membrane (MS) was statistically equal to the modulus of the support layer, the average values of which were 384 and 398 MPa, respectively. The non-supported membrane presented a Young modulus of 76 MPa, about five times smaller than those of the support layer and the supported membrane, indicating a smaller resistance to deformation of the non-supported membrane, relative to the supported one.

Pavoni et al. [15] reported a Young modulus of 1912 MPa for chitosan membranes crosslinked with 5 wt % glutaraldehyde, along with a grammage of 0.34 g∙m^−2^, Liu et al. [58] observed that for a membrane crosslinked with 1 wt % glutaraldehyde, a Young modulus was recorded of 450 MPa, whereas Priyadarshi et al. [59] reported a Young modulus of 42.8 MPa and 3221.2 MPa for chitosan films without and with crosslinking, respectively. These results suggest great variability in mechanical properties that can be attributed to the different parameters used in the production of chitosan membranes, for instance, the acid and crosslinker types and concentrations, the degree of deacetylation, and the viscosimetric molar mass of chitosan. The cure parameters, such as temperature and time, can also influence the mechanical properties of the final membranes [22,60].

According to Clasen et al. [61], chitosan membranes with a Young modulus that is higher than 755 MPa may be undesirable for some applications because it becomes too rigid and fragile, fracturing easily. PVC films that are used as food packaging have an average Young modulus of 45 ± 6 MPa [15]; thus, it is possible to suggest the comparison that the non-supported membrane (M) had a flexibility and mechanical resistance similar to the commercial PVC films; the supported membrane (MS) has a higher rigidity due to the support layer of PET.

The tensile strength of the supported membrane and the support layer were statistically similar; with 26.5 MPa and 27.0 MPa, respectively, showing the same behavior of the Young modulus. The non-supported membrane has a tensile strength of 16.3 MPa, which is about 38% smaller, relative to the supported one. The higher tensile strength of the supported membrane was due to the presence of the support layer associated with a greater global thickness, which increases the mechanical resistance of the supported membrane when compared to the non-supported one.

The values of tensile strength also presented a wide variation, as presented in different studies. Pavoni et al. [55] reported a tensile strength of 38 ± 3 MPa for chitosan membranes, crosslinked with 5 wt % glutaraldehyde. Liu et al. [62] observed a tensile strength of 67 ± 1 MPa for chitosan membranes crosslinked with 1 wt % glutaraldehyde, and Priyadarshi et al. [59] reported tensile strengths of 9.5 MPa and 52.2 MPa for chitosan films without and with crosslinking, respectively.

The PET support layer presented an elongation at break of 29.9%, being statistically similar to the supported membrane (26.1%). The elongation at break was smaller for the non-supported membrane (6.5%), indicating the low malleability (high fragility) of the membrane when not supported.

Frick et al. [34] and Pavoni et al. [15] reported elongations at break of 7 ± 2% for chitosan membranes crosslinked with 5 wt % glutaraldehyde, whereas the elongation for non-crosslinked membranes, produced under the same conditions, was 45 ± 3%. Liu et al. [62] reported that for chitosan membranes crosslinked with 1 wt % glutaraldehyde, elongation would be at a break of 12 ± 1%. Priyadarshi et al. [59], evaluating the properties of membranes without and with crosslinking, observed a reduction of the elongation at break from 26.4% in the non-crosslinked membrane, to 2.3% in the crosslinked one; however, these authors also used glycerol as a plasticizer.

Beppu et al. [56] and Frick et al. [34] cited the effect of crosslinking on the elongation at break of the films and membranes of chitosan, in which the elongation at break decreases as the concentration of the crosslinker increases. This happens due to the immobilization of the polymeric chains of chitosan, reducing the overall elasticity and increasing the rigidity of the crosslinked material, compared to the one not crosslinked.

Thus, the use of a support layer increased the mechanical properties of the membrane, one the non-supported membrane may present itself, as being excessively malleable, or even breaking easily. The use of a support layer increased the resistance to breakage, malleability, and the flexibility of the supported membrane.

#### 3.2.3. Contact Angle and Surface Energy

The contact angle of the membranes with distilled water, diiodomethane, ethanol 96%, linalool, and myrcene was measured; these substances were chosen due to their different polarity degrees and the kind of interaction with other surfaces. The results are compiled in Table 2.

According to the results of contact angle, it can be observed that the presence of the support layer has caused no difference relative to chemical affinity since, for all tested substances, the contact angles were similar, not differing statistically. The exception was the linalool, whose contact angle presented a statistical difference between the non-supported (zero) and the PET-supported membrane (14°). This may also indicate that the interaction between the chitosan active layer and the support layer is physical, with no chemical reaction occurring between the layers [15].

Relative to the contact angle between the water and the membranes, the observed values (85–88°) suggest that the membranes have a more hydrophobic characteristic. Considering the diiodomethane, whose interactions are almost exclusively of the dispersive type, this behavior was distinctive, wherein the support layer presented the lowest contact angle (27°), followed by the non-supported membrane (36°), and the supported membrane (44°). A possible explanation of the difference in the contact angles of the membranes with diiodomethane may be the effect of the crosslinker (glutaraldehyde) on the chitosan structure. However, it is not possible to infer the degree of interaction of the membranes with polar or non-polar substances, only via an evaluation of the contact angle [63].

The support layer had a contact angle of zero in the case of ethanol, linalool, and myrcene; this indicates the high affinity of this polymer with the tested substances. However, it is also important to highlight the smaller contact angle of this polymer with water (63°), when compared to the membranes (85–88°). According to Prajitno et al. [64], rougher surfaces that have an affinity with a substance tend to have lower contact angles than the smoother surfaces of the same material.

Zhang et al. [57] reported that for chitosan membranes crosslinked with 2 wt % glutaraldehyde, superficially modified by immersion in acetone, the contact angle for water was recorded as 97 ± 1°. Shenvi et al. [65], working with chitosan membranes crosslinked with 0.2 wt % and 0.6 wt % glutaraldehyde, reported contact angles with water of 73.2° and 79.9°, respectively. The membranes produced by Pavoni [15], with different concentrations of glutaraldehyde as a crosslinker, showed contact angles for water in the range of 89–93°.

Considering the measured contact angles between the membranes and the other substances (ethanol, linalool, and myrcene), both membranes presented quite similar behavior, where the contact angles were smaller than 40°. Relative to the substances, ethanol presented a slightly higher angle (33° and 31° for M and MS) than myrcene (24° for both membranes). The observed behavior for linalool was different, in that both the support layer and the non-supported membrane had a contact angle of zero, whereas the supported membrane presented an angle of 14°. This may indicate a smaller affinity between the linalool and the supported membrane. However, it is important to consider that this contact angle (14°) was the smallest among all the substances tested for this membrane, following the pattern observed for the non-supported and the support layer. The higher contact angle observed for the supported membrane may also have arisen due to variations in membrane production.

Pavoni [15] reported contact angles between the membranes and ethanol in the range of 18–23°, suggesting a higher “wetting” of the membrane surface by ethanol, whereas water presented a smaller degree of spreading, with contact angles near 90°. Thus, it could be considered that this membrane may have a higher affinity with non-polar or slightly polar substances. On the other hand, in the case of chitosan membranes crosslinked with glutaraldehyde, Clasen et al. [61] reported a contact angle with mercury of 141.3°. However, according to Jasper [66], mercury has a high surface tension (425.4 mN∙m^−1^) when compared to ethanol (22.1 mN∙m^−1^), terpenes in general (20–30 mN∙m^−1^), diiodomethane (50.8 mN∙m^−1^), or even water (72.1 mN∙m^−1^).

The calculated surface energies and the dispersive and polar components for the membranes and the support layer, determined by the Fowkes method, are presented in Table 3.

According to Table 3, the calculated surface energy of the non-supported membrane (55 mJ∙m^−2^) was similar to the supported membrane (49 mJ∙m^−2^), whereas the surface energy of the support layer was approximately 1.5 times higher (81 mJ∙m^−2^). According to Law [63] and Jańczuk and Białlopiotrowicz [67], surfaces and liquids with similar surface energies tend to have lower contact angles than systems that have greater differences in their surface energies.

Beyond the total surface energy (σ), it is also important to consider that this is a summation of its respective dispersive (σ^D^) and polar (σ^P^) components [42]. The membranes and the support layer presented higher σ^D^ in the range of 35–45 mJ∙m^−2^, indicating a stronger effect of the dispersive interactions, which have a non-polar characteristic (van der Waals forces). However, while the membranes presented small σ^P^ values in the range of 10 mJ∙m^−2^, the support layer presented σ^P^ values that were three times higher (36 mJ∙m^−2^), which may explain the smaller contact angle of the support layer with water when compared to the angles between water and the membranes. Nevertheless, it is important to consider that polymer rugosity may influence the spreading of the liquid at the solid–liquid interface [63,66].

The σ^D^ of the membranes was in the range of 35–45 mJ∙m^−2^, which was similar to the surface tension of ethanol and myrcene (20–30 mN∙m^−1^). It could also be observed that the support layer presented a high affinity for ethanol and myrcene, with zero contact angle, and also has a higher affinity for water (a contact angle of 63°). It is important to consider that the units of surface tension and surface energy are dimensionally equivalent (J·m^−2^ = N·m^−1^ = kg·s^−2^). Thus, substances with similar surface tensions, such as ethanol and myrcene, had similar contact angles (in the range of 20–35°) with both membranes, whereas water, which has a strong polar component of surface tension, presented a higher contact angle (near 90°). The support layer also presented a higher σ^P^, which may explain a stronger interaction with water. However, the support layer also interacted with ethanol, linalool, and myrcene under the same conditions, but this may arise because of the porosity of the support layer, which enhances the interaction between the surfaces due to capillary effects [63].

The greater proximity between the surface energies of the membranes and the compounds myrcene, linalool, and ethanol may explain the lower contact angle since great differences in surface energy/tension, as well as the surface energy components, will cause repulsive effects between the interfaces [63,66,67].

#### 3.2.4. Swelling Degree

The swelling degree of the membranes was determined using distilled water, ethanol at 96% vol., linalool, and myrcene, aiming to verify the possible sorption patterns of both membranes. The results relative to the mass and volumetric swelling degree are presented in Table 4.

Relative to the swelling degree, it was possible to observe that both membranes absorbed water profusely. The non-supported membrane (M) presented an increase in mass of more than three times the initial value, due to water absorption during the 24 h of the test. However, the supported membrane (MS) increased its mass by approximately 50% because the PET support layer had not absorbed water and the mass of the PET layer was greater than that of the chitosan layer. The same behavior was observed in the volumetric (thickness) swelling, considering that the non-supported membrane presented a thickness increase of more than two times. The supported membrane presented a volumetric swelling degree of more than 37%. However, due to the presence of the support layer, this membrane presented a smaller volumetric swelling but was still quite expressive, considering that the ratio between the thickness of the entire membrane and the chitosan layer is 4.2 (119.6 µm and 28.2 µm, respectively).

The crosslinking of chitosan films/membranes is necessary for the films/membranes to have some degree of resistance to water. Due to the high degree of affinity between chitosan and water, the fast diffusion of water occurs throughout the membrane structure, inducing high degrees of swelling [15,22,23,60].

Liu et al. [62] reported a mass swelling degree of 110% after 24 h of immersion in water for chitosan membranes crosslinked with 2 wt % glutaraldehyde. Pavoni et al. [15,55] reported in two separate studies, for chitosan membranes crosslinked with 5 wt % glutaraldehyde and immersed in water, swelling degrees of 571 ± 105% and 697 ± 195%, respectively. The same authors reported a volumetric (thickness) swelling degree of 145% and 210%, respectively.

The performance of supported and non-supported membranes when exposed to ethanol was quite similar for the mass swelling, although the behavior was inverse relative to the volumetric swelling. Both membranes presented small variations (1.0% and zero, respectively) in mass swelling after exposure to ethanol, indicating that this substance was sorbed and diffused through the membranes at quite small rates. Regarding the volumetric swelling, it was observed that the non-supported membrane had an average expansion of 4.6%, whereas the supported membrane had a contraction of 7.9%. A possible explanation for this phenomenon may be related to the interaction and diffusion between ethanol and the PET support layer, changing its structure and even causing compaction [22,23]. The slight increase in the thickness of the non-supported membrane may be the result of sorption and the diffusion of small amounts of ethanol, which may have caused a separation of the polymeric chains of chitosan, consequently increasing membrane thickness [68].

For linalool and myrcene, these induced mass swelling degrees of approximately 20% and 15%, respectively, for both membranes. This indicates that these substances were sorpted and diffused through the membranes to an extent. By analyzing the volumetric swelling, it was possible to notice that both the linalool and myrcene were sorpted by the non-supported membrane (M) since its thickness increased by 8.4% when exposed to linalool and 8.8% when exposed to myrcene. Curiously, there was a contraction of 3.4% of the PET-supported membrane when exposed to myrcene, due to interaction effects between this substance and the PET layer, which may have caused a partial rearrangement of the polymeric chains of the supporting polymer [15]. However, the volumetric swelling degree for linalool was positive (6.1%), indicating that, possibly, the linalool was adsorbed and diffused in both the chitosan and the PET layers, although only to a small extent.

Considering the small contact angle (20° to 35°) and the relatively small swelling degree of the membranes when exposed to ethanol (0.0–1.0%), linalool, and myrcene (13–19%), the obtained membranes will probably show chemical resistance and reasonable performance in the pervaporation process that involves these types of substances. High swelling degrees (> 30%) tend to destabilize the membrane structure during the pervaporation process, hindering transmembrane diffusion and reducing membrane selectivity; high contact angles (> 90°) may indicate incompatibility between the substance that must permeate and the membrane, impairing the sorption process [15,69].

### 3.3. SEM Analysis and Membrane Microstructure

**Figure 5:**
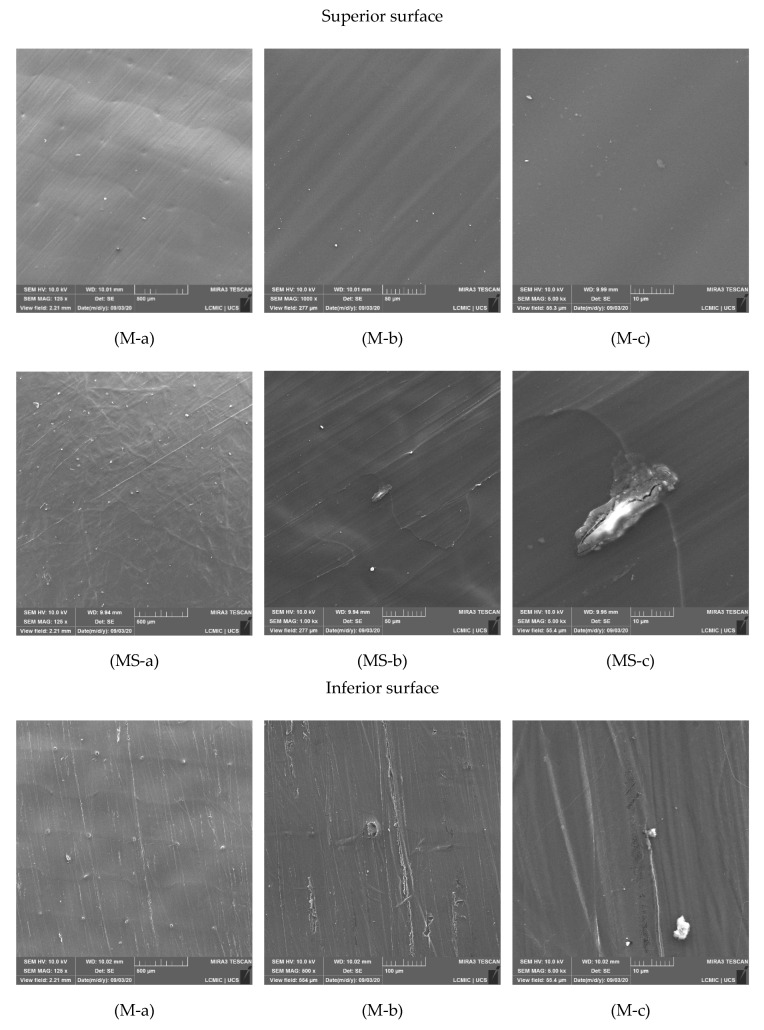

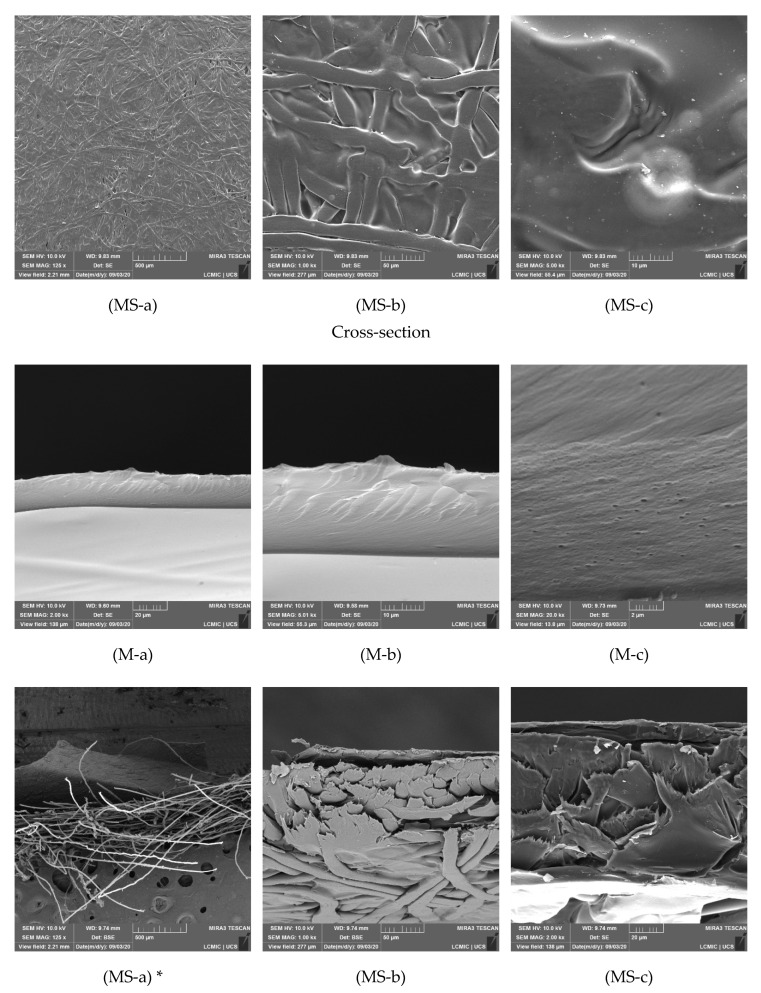
Micrographs of the superior and inferior surfaces and the cross-sections of the active layers of the non-supported (M) and PET-supported (MS) chitosan membranes, at magnifications of 125 (**a**), 1000 (**b**), and 5000 (**c**) times. *—The cross-section of this membrane was obtained via cryogenic fracture; the cross-sections of the other MS membranes were obtained by cutting using a scalpel.

The morphological aspect of each membrane was evaluated. The micrographs of both membranes, at different magnifications, are presented in Figure 5.

Observing Figure 5 (M-a to M-c), a high degree of homogeneity of the air-exposed surface of the non-supported membrane is visible. Most of the surface was smooth, with a small number of defects, and a homogeneous covering.

The micrographs of the active layer of the PET-supported membrane (Figure 5, MS-a to MS-c) showed a surface that was mostly smooth. However, there are more defects in it than in the non-supported membrane. Although small, it was possible to observe the presence of granules that, when magnified in 5000× (Figure 5, MS-c), appeared to be small fragments of chitosan (with a size of about 15–20 µm) that have not dissolved completely, even after the vigorous stirring of the polymeric solution for 24 h.

It was also noteworthy that at a magnification of 125× (Figure 5 MS-a), it was possible to observe the microstructure of the support layer below the active layer, similarly to the marks left by the Teflon^®^ layer in the pure (non-supported) membrane. It was also possible to see a complete spreading of the active layer of chitosan on the support layer. Pavoni [15] reported a different behavior, in which the active layer of chitosan in some of the produced membranes has not completely coated the support layer.

Haghighi et al. [19], studying the use of essential oils as additives in the production of blended gelatin-chitosan films, also reported the formation of a smooth and homogeneous surface. Al-Naamani et al. [70], applying chitosan to polyethylene films, reported the formation of a well-distributed and homogeneous film with almost no defects. Zhang et al. [57], who produced chitosan membranes crosslinked with glutaraldehyde, superficially modified by immersion in acetone, also obtained membranes with smooth surfaces and no important defects.

Regarding supported membranes, Shi et al. [71] developed composite chitosan-polyacrylonitrile (PAN) membranes and observed that by increasing the chitosan concentration in the polymeric solution, the active layer tended to become thicker. For the concentration of 3 wt % of chitosan, the active layer coated the supporting layer. However, only for 5 wt % of chitosan, the support layer was entirely covered, not being possible to detect the support layer in the micrographs. Kazemi et al. [72] produced a hybrid multilayer membrane of chitosan and alginate, with the addition of WO_3_ nanoparticles as carriers. The authors observed a complete and homogeneous coating of the chitosan layer on the entire surface of the substrate.

Micrographs of the surface of the non-supported membrane that was in contact with the Teflon^®^ layer during the curing process, as well as the PET surface of the supported membrane, are presented in Figure 5. From Figure 5 (Inferior surface, M-a to M-c), it is possible to observe that the membrane side in contact with the Teflon^®^ presented a rougher surface, with some defects. These may be the result of the detachment of the membrane from the Teflon^®^ layer, which may generate defects in the surface when the membrane is removed from the Petri dish. In addition, the presence of irregularities in both the Teflon^®^ layer and the Petri dish itself may have been imprinted in the produced membranes.

The surface of the PET layer of the supported membrane was heterogeneous, composed of fibers disposed in a random pattern in an amorphous matrix (Figure 5, Inferior surface, MS-a to MS-c). This structure is characteristic of non-woven composite polymers, produced by the partial melting of the amorphous part and of the fibers, which agglomerated and amalgamated [73,74].

Guerrero et al. [75], working with chitosan membranes crosslinked with citric acid and produced by compression molding, reported rough surfaces with many defects. However, the same authors cited that the use of larger quantities of citric acid (10 wt % and 20 wt %, relative to chitosan mass) reduced the number of surface defects. Acosta et al. [76], carrying out experiments using starch-chitosan films, observed the presence of surface defects in the films produced; the defects were mostly cavities and microbubbles.

Iranizadeh et al. [77], Terraza et al. [73], and Wang et al. [78], working with thin-film composite membranes deposited on non-woven supports of polyester, PET, and PVC, reported similar microstructures, with the presence of randomly disposed fibers spread over a mostly amorphous matrix. According to these authors, the support has intrinsic porosity due to the production method, generating layers with moderate porosity. The presence of pores may help in the permeation process, unlike dense support layers, which, due to the existence of an additional diffusion stage, may end up hindering permeation in general.

It was also possible to verify that the support layer remained unchanged during the curing of the membrane, indicating that both the curing process and the contact with the polymeric solution do not have any effect on the support layer of PET. Thus, the physical-chemical properties of this layer that are important for the pervaporation process (contact angle, surface energy, and porosity) most likely were not changed.

The micrographs of the cross-section of the non-supported and PET-supported membranes at different magnifications are reported in Figure 5. According to the micrographs of the cross-section of the non-supported membranes, it was possible to verify that they were dense, with a small number of microbubbles and defects, as can be seen in Figure 5 (M-c). There are microbubbles because micropores span throughout the entire thickness of the active layer. The microbubbles might be the result of the stirring of the polymeric solution; they were not removed during the resting time, probably due to the high viscosity of the solution.

Analyzing Figure 5 (Cross-section, MS-a), it was possible to observe that the cross-section obtained by cryogenic fracture had a ‘disheveling’ effect, i.e., the fibers of the support layer were separated from the amorphous matrix, generating a chaotic and disorganized system. It was also possible to see in the other micrographs (Figure 5, Cross-section, MS-b and MS-c) that the active layer presented a homogeneous thickness throughout the entire cross-section. This behavior indicated that the chitosan has presented a good spread and mechanical adherence to the support layer, without the occurrence of intrusions of the active layer in the support layer, a phenomenon that is harmful to membrane performance which is common in membranes produced by casting [7,8].

Clasen et al. [61], who studied the formation of chitosan films and membranes by crosslinking with glutaraldehyde, obtained dense membranes with a cross-section presenting a small number of bubbles and defects. Zhang et al. [57], who developed a chitosan membrane for hydrophilic pervaporation, reported the production of dense membranes with a homogeneous cross-section and without apparent defects. On the other hand, Guerrero et al. [75] obtained chitosan membranes crosslinked with citric acid with an irregular cross-section, presenting granular and heterogeneous characteristics; pores and defects were observed along the entire extension of the cross-section. However, it is important to consider that these membranes were produced via mold compression and not by casting and solvent evaporation.

The dense character of the membrane is important for the pervaporation process, a membrane with dense characteristics and a homogeneous cross-section tends to have more uniform diffusion throughout the entire surface and cross-section than in heterogeneous membranes where the sorption and diffusion rates may tend to differ, impairing membrane efficiency and, consequently, the pervaporation process as a whole [8,22,57].

Pavoni [15] reported the development of a chitosan membrane crosslinked with glutaraldehyde and supported on PET, where the cross-section, obtained by cutting, was composed of a thin active layer adhered to the support layer. Chrzanowska et al. [26], studying the production of chitosan membranes supported on polyamide-6, reported that the support layer remained adhered to the support layer but without the presence of intrusions. Moulik et al. [32] also observed a similar behavior, as observed in this study, for chitosan membranes crosslinked with tetraethyl orthosilicate (TEOS) and supported on polytetrafluoroethylene (PTFE).

Shenvi et al. [65], investigating the production of chitosan membranes supported on poly (1,4-phenylene-ether-ether-sulfone) (PPEES), reported the presence of intrusions of the active layer into the support; the authors attributed this phenomenon to the morphology of the PPEES, which was composed of cavities, mainly in the longitudinal direction of the thickness. Kononova et al. [79], working on the development of a hybrid chitosan membrane supported on a polyelectrolyte complex, reported the formation of a homogeneous active layer without the occurrence of intrusions, citing that the physical-chemical characteristics of the support have a fundamental role in the formation or absence of interlayer intrusions.

### 3.4. Pervaporation Tests

Target-organophilic and hydrophilic pervaporation tests were carried out with both the PET-supported and the non-supported membranes. Regarding target-organophilic pervaporation, no permeate was collected even after 8 h of the process for all membranes.

At first sight, this may indicate a low degree of affinity between the feed components (limonene and linalool) with chitosan, which would hinder or even prevent the sorption and diffusion process. However, the swelling degree tests (Table 4) showed that there was an increase in the mass of the membranes when they were exposed to limonene and linalool, indicating that the sorption and diffusion process occurred, albeit slowly.

According to Mulder [7], Baker et al. [44], and Sahin [69], the driving force of the separation in pervaporation is the chemical potential difference between the membrane sides; the main parameter that can be controlled, which influences the chemical potential, is the pressure of permeate stream. The same authors also commented that the pressure difference that must be considered to be the driving force is not an absolute difference; instead, it should be addressed from a thermodynamic standpoint (chemical potential). In this sense, the real pressure difference between membrane sides is related to the saturation pressure of the liquid components of the feed stream and the partial pressure of the components in the vapor phase of the permeate.

In the experiments, the temperature and the permeate pressure were kept at 40 °C and 40 Torr (5.33 kPa), respectively. Considering feed composition, the vapor saturation pressure (P^sat^) for limonene at 40 °C is 5.0 Torr (665 Pa) and for linalool, it is 0.6 Torr (approx. 80 Pa), not considering Raoult’s law (the reduction of effective saturation pressure due to dilution) and possible deviations from ideality [80,81]. In general, the pressure range in permeate stream for target-organophilic pervaporation is quite low, below 10 Torr (1.33 kPa). As addressed by Moulik et al. [32], an increase in permeate pressure may severely reduce the transmembrane flux and the separation factors, due to the reduction and/or reversal of the driving force (chemical potential difference) responsible for the separation. It is also important to point out that in studies of both hydrophilic and organophilic pervaporation, the absolute pressures of permeate stream are very low, generally below 500 Pa (3.75 Torr) [7,32,82].

Formally following the modified Raoult’s law (P_i_^eff^ = x_i_·γ_i_·P^sat^) using the calculated activity coefficients data (γ_limonene_ = 1.1218 and γ_linalool_ = 1.9377 at 40 °C and the molar fractions of x_limonene_ = 0.7746 and x_linalool_ = 0.2254, respectively), the effective saturation pressures of limonene and linalool in liquid (feed) stream would be 580 Pa and 35 Pa, respectively. Considering that thermodynamic equilibrium occurs when the partial pressures of the compounds equal the effective pressures in the liquid phase, this implies partial pressures of the limonene and linalool of 580 Pa and 35 Pa in the permeate stream. According to Dalton’s law, 5330 Pa is the absolute pressure of permeate stream; the molar fractions of limonene and linalool in the permeate in the equilibrium would then be 0.109 and 0.007, respectively. Moreover, considering that the chitosan membrane has a polar characteristic and, therefore, has less affinity with limonene, this could explain the lack of measurable permeate due to the low molar fraction of linalool in the permeate stream.

On the other hand, permeate was collected in the hydrophilic pervaporation tests. Water and ethanol saturation pressures at 40 °C were 55.40 Torr (7.38 kPa) and 134.33 Torr (17.91 kPa), respectively [80,83], being higher than the pressure of permeate stream (40 Torr—5.33 kPa). Unlike the results with limonene and linalool in target-organophilic pervaporation, the chemical potential was higher in the feed stream, thus directing the mass transfer of both ethanol and water from the feed side to the permeate side of the membrane.

Thus, membrane affinity played the role of separating the two substances by differential sorption and diffusion of the species throughout the membrane, which occurred at different rates for each substance [44,84]. Data for the hydrophilic pervaporation are presented in Table 5.

According to the data presented in Table 5, with the exception of PSI (the pervaporation separation index), all other parameters differed statistically between non-supported and supported membranes. In pervaporation processes, transmembrane flux (J), selectivity (α), PSI, and permeate composition are regarded as the most important parameters when considering membrane performance [7,13,44].

It is important to observe that transmembrane fluxes, permeances, and permeabilities for both water and ethanol were higher in the non-supported membrane. According to Trifunovic and Trägardh [85] and Eljaddi et al. [86], the presence of a support layer directly impacts the mass transfer because the addition of a new layer, albeit porous, also poses resistance to mass flux through Knudsen diffusion (tortuous diffusion). The authors also commented that a thin and highly porous support layer may help to mitigate mass transfer reduction through the presence of a greater area composed of pores, acting as a “support-free” region, as well as a reduction in the tortuous path for the diffusing molecules.

Regarding transmembrane flux (J), this value was about three times higher in the non-supported membrane (0.397 kg·m^−2^·h^−1^) when compared to the PET-supported membrane (0.121 kg·m^−2^·h^−1^). In both membranes, water flux was much higher than ethanol flux (water/ethanol flux ratios of 20.1 and 50.5 for the non-supported and PET-supported membrane, respectively), indicating a greater membrane affinity with water. This was expected, considering that chitosan membranes are widely employed in hydrophilic pervaporation [22,33].

It has been reported that the performance of composite chitosan membranes is supported on various materials. Fini et al. [29], working with chitosan membranes supported on α-alumina and mullite-alumina, reported transmembrane fluxes in the range of 0.18–0.35 kg·m^−2^·h^−1^. Pavoni [15], studying the pervaporation performance of chitosan membranes crosslinked with glutaraldehyde at 50 °C and an ethanol feed concentration of 10% v/v, reported a transmembrane flux of 5.40 kg·m^−2^·h^−1^ (5.40 L·m^−2^·h^−1^); the water/ethanol flux ratio was 7.3. Ma et al. [87] and Wang et al. [88] reported, using membranes composed of chitosan as the active layer and polyacrylonitrile (PAN) as a support layer, transmembrane fluxes of 0.60 kg·m^−2^·h^−1^ and 0.26 kg·m^−2^·h^−1^, respectively.

Separation (or enrichment) factors (β) were also influenced by the presence of the support layer, increasing from 3.17 in the non-supported membrane to 3.28 in the supported one; ethanol had an inverse trend, decreasing from 0.07 to 0.03 for non-supported and supported membranes, respectively. This reduction in β for ethanol may be the result of the smaller transmembrane flux, which is inversely related to membrane selectivity. In this sense, the support layer, having a greater affinity with ethanol, may have hindered its permeation by a possible accumulation of this substance relative to water, which was promptly removed due to the hydrophobicity of PET [85].

Regarding the literature, separation factors are also a function of feed composition, so there is great variability of results when considering this parameter [22,84]. Fini et al. [29] reported separation factors for chitosan-PAN composite membranes, ranging from 130 to 200 as ethanol feed concentration increased from 50 wt % to 90 wt %; at a concentration of 70 wt %, the separation factor was 180. Kang et al. [89] reported for chitosan membranes crosslinked with glutaraldehyde a separation factor of 148. Chrzanowska et al. [26] studied the performance of a chitosan membrane on a feed composed of 90 wt % ethanol and 10 wt % water and reported a separation factor of 18 ± 2; the addition of a polyamide-6 support layer increased the separation factor to 39 ± 1.

For both membranes, water permeability (P) and permeance (P’) were higher than for ethanol. However, the non-supported membrane presented statistically higher permeances and permeabilities for both substances relative to the PET-supported membrane. Dharupaneedi et al. [90] reported a total membrane permeance value of 6.76·10^−13^ kg·m^−2^·s^−1^·Pa^−1^ for a water feed concentration of 10 wt % and a feed temperature of 40 °C. Dudek et al. [91] reported, for glutaraldehyde-crosslinked membranes used in the pervaporation of a hydroalcoholic solution containing 50 wt % ethanol and a permeate pressure of 300 Pa, water and ethanol permeabilities of 6.87·10^−8^ and 1.06·10^−8^ kg·m^−1^·s^−1^·Pa^−1^, respectively.

Membrane selectivity (α) was also influenced by the presence of the support layer, increasing from 611, in the non-supported membrane, to 1974 in the PET-supported one. This increase in selectivity may have occurred due to the same effect that causes the reduction in transmembrane flux, in which a hydrophobic support layer (such as the one used in this work) may help to foster water mass transfer via repulsive interactions between the support (PET) and the permeating water, forcing the latter out of the support structure and reducing its accumulation downstream the membrane. However, interface areas between the support and the active layer of chitosan will not be available to permeate the water, reducing the effective permeation area and thereby reducing the overall transmembrane flux [85,86].

There is some divergence in the literature when addressing membrane selectivity because several works regard membrane selectivity and separation factors as similar parameters; indeed, there are several works that regard these parameters as the same. However, as highlighted by Baker et al. [44] and Mulder [7], the optimal approach is by treating selectivity as an intrinsic membrane property, i.e., as the ratio between permeances (or permeabilities), rather than the ratio between enrichment factors, which depend on feed composition.

Dharupaneedi et al. [90] reported a water/ethanol selectivity of 171 for a water feed content of 30 wt % for chitosan membranes crosslinked with glutaraldehyde. On the other hand, Dudek et al. [91] reported a chitosan membrane crosslinked with glutaraldehyde with a water/ethanol selectivity of 6.5. Sunitha et al. [92] produced a phosphorylated chitosan membrane with a selectivity of 183 when used in the pervaporation of an ethanol/water solution with a water content of 10.23 wt % at 30 °C and permeate pressure of 0.5 Torr (67 Pa). Zielinska et al. [93] reported for a crosslinked chitosan hydrogel membrane with a water/ethanol selectivity of 27 for a water feed content of 10 wt %.

The PSI was the only parameter that has not differed between membranes, ranging between 218 and 228 for the PET-supported and the non-supported membranes, respectively. Considering that PSI is a generalized approximation by which to quantify membrane performance, based on membrane selectivity and transmembrane flux, despite the quite different selectivities and fluxes, both membranes, overall, had a similar performance [10,94]. In this sense, both membranes could be regarded as similar; however, a more in-depth analysis of membrane performance relative to selectivity or transmembrane flux may be required to determine what kind of membrane may be more suitable for a specific application.

## 4. Conclusions

Although the presence of the PET layer has presented a negligible effect on most of the physical–chemical parameters of the membrane, the mechanical parameters were influenced and the presence of the support layer increased membrane mechanical resistance. Both membranes were unsuitable for application in target-organophilic pervaporation, considering the operating conditions, used since no permeate was obtained, regardless of the presence or absence of the PET layer. To render these membranes suitable for target-organophilic pervaporation, process conditions need to be more stringent, especially regarding the pressure of the permeate stream. However, the support layer influenced membrane performance in hydrophilic pervaporation, in which the support layer increased membrane selectivity and reduced permeability and permeance for both water and ethanol. This was likely due to an increase in the overall hydraulic resistance of the membrane, with even the support layer being porous. Moreover, the PSI has not differed between membranes, indicating that a more in-depth analysis linked to specific applications is necessary regarding individual membrane performance.

## Figures and Tables

**Table 1:** Mechanical properties of the support layer (PS) and the supported (MS) and non-supported (M) membranes.

Parameter	PS	M	MS	CV * (%)
Thickness (µm)	91 ± 2 ^b^	20 ± 1 ^c^	120 ± 2 ^a^	2.5
Young modulus (MPa)	384 ± 40 ^a^	76 ± 7 ^b^	398 ± 18 ^a^	8.9
Tensile strength (MPa)	27 ± 9 ^a^	16 ± 1 ^b^	27 ± 3 ^a^	22.2
Elongation at break (%)	30 ± 4 ^a^	7 ± 1 ^b^	26 ± 3 ^a^	15.1

Means in rows followed by the same letter do not present a statistical difference, as measured by Tukey’s multiple range test at 5% error probability (α = 0.05). * —CV: coefficient of variation.

**Table 2:** Contact angle (°) of the PET-supported (MS) and non-supported (M) membranes and the support layer (PS) for distilled water, diiodomethane, ethanol 96%, linalool, and myrcene (CV = 5.93%).

Substance	Contact Angle (°)		
	M	MS	PS
Distilled water	85 ± 1 ^a^	88 ± 3 ^a^	63 ± 2 ^b^
Diiodomethane	36 ± 1 ^b^	44 ± 1 ^a^	27 ± 1 ^c^
Ethanol 96% v/v	33 ± 3 ^a^	31 ± 3 ^a^	0 ± 0 ^b^
Linalool	0 ± 0 ^b^	14 ± 1 ^a^	0 ± 0 ^b^
Myrcene	24 ± 3 ^a^	24 ± 3 ^a^	0 ± 0 ^b^

Means in line followed by the same superscript letter do not differ by Tukey’s multiple range test at 5% error probability (α = 0.05).

**Table 3:** Surface energy (σ) and the dispersive (σ^D^) and polar (σ^P^) components for the PET-supported (MS) and non-supported (M) membranes and the support layer (PS), determined using the Fowkes method.

Material	Surface Energy (mJ∙m^−2^)		
	Total (σ)	Dispersive (σ^D^)	Polar (σ^P^)
M	55 ± 1	42 ± 1	13 ± 1
MS	49 ± 1	38 ± 2	12 ± 1
PS	81 ± 3	45 ± 3	36 ± 3

**Table 4:** Mass and volumetric (thickness) swelling degree of the PET-supported (MS) and the non-supported (M) membranes after 24 h of exposure to distilled water, ethanol 96% vol., linalool, and myrcene.

Liquid	Mass Swelling (%)		Volumetric Swelling (%)	
	M	MS	M	MS
Distilled water	318.1 ± 32.5 ^a^	53.3 ± 10.2 ^a^	229.3 ± 10.5 ^a^	37.3 ± 4.6 ^a^
Ethanol 96% v/v	1.0 ± 2.0 ^c^	0.0 ± 0.0 ^c^	4.6 ± 2.8 ^b^	− 7.9 ± 2.2 ^c^
Linalool	19.3 ± 4.5 ^b^	16.9 ± 4.5 ^b^	8.4 ± 0.7 ^b^	6.1 ± 1.0 ^b^
Myrcene	14.1 ± 2.0 ^b^	13.0 ± 0.7 ^b^	8.8 ± 2.8 ^b^	− 3.4 ± 1.8 ^c^

Means that are followed by the same superscript letter in the column labeled “liquid” do not differ statistically by Tukey’s multiple range test at 5% error probability (α = 0,05). The coefficient of variation was 6.24% for mass swelling and 11.38% for volumetric (thickness) swelling, respectively.

**Table 5:** Pervaporation parameters for the PET-supported and the non-supported membranes in the hydrophilic pervaporation of the ethanol/water mixture (70/30 wt %).

Parameter	Unit	Non-Supported Membrane	PET-Supported Membrane	Coefficient of Variation (%)
Transmembrane flux (J)	kg·m^−2^·h^−1^	0.397 ^a^	0.121 ^b^	5.3
Water flux (J_W_)	kg·m^−2^·h^−1^	0.378 ^a^	0.119 ^b^	4.7
Ethanol flux (J_E_)	kg·m^−2^·h^−1^	0.019 ^a^	0.002 ^b^	8.4
Water content in permeate (C_W,P_)	wt %	95.2 ^b^	98.3 ^a^	0.2
Ethanol content in permeate (C_E,P_)	wt %	4.77 ^a^	1.74 ^b^	21.0
Water enrichment factor (β_W_)	-	3.17 ^b^	3.28 ^a^	3.1
Ethanol enrichment factor (β_E_)	-	0.07 ^a^	0.03 ^b^	10.3
Water permeance (P_W_’)	kg∙m^−2^∙s^−1^∙Pa^−1^	5.61∙10^−7 a^	1.76∙10^−7 b^	19.0
Ethanol permeance (P_E_’)	kg∙m^−2^∙s^−1^∙Pa^−1^	9.77·10^−10 a^	1.13·10^−10 b^	23.2
Water permeability (P_W_)	kg·m^−1^·s^−1^·Pa^−1^	1.68∙10^−11 a^	3.52∙10^−12 b^	11.3
Ethanol permeability (P_E_)	kg·m^−1^·s^−1^·Pa^−1^	2.93·10^−14 a^	2.25·10^−15 b^	18.3
Selectivity (α_W/E_)	-	611 ^b^	1974 ^a^	10.6
PSI ^1^	kg·m^−2^·h^−1^	228 ^a^	218 ^a^	11.1

Means in rows followed by the same superscript letter do not present statistical difference by Tukey’s multiple range test at 5% error probability (α = 0.05). ^1^ —Pervaporation Separation Index.

## Data Availability

All data used in this study is presented in the article.
